# Healthcare Workers’ SARS-CoV-2 Omicron Variant Uncertainty-Related Stress, Resilience, and Coping Strategies during the First Week of the World Health Organization’s Alert

**DOI:** 10.3390/ijerph19041944

**Published:** 2022-02-09

**Authors:** Mohamad-Hani Temsah, Shuliweeh Alenezi, Mohammed Alarabi, Fadi Aljamaan, Khalid Alhasan, Rasha Assiri, Rolan Bassrawi, Fatimah Alshahrani, Ali Alhaboob, Ali Alaraj, Nasser S. Alharbi, Abdulkarim Alrabiaah, Rabih Halwani, Amr Jamal, Naif Abdulmajeed, Lina Alfarra, Wafa Almashdali, Ayman Al-Eyadhy, Fahad AlZamil, Sarah Alsubaie, Mazin Barry, Ziad A. Memish, Jaffar A. Al-Tawfiq

**Affiliations:** 1 College of Medicine, King Saud University, Riyadh 11362, Saudi Arabia; salenizi@ksu.edu.sa (S.A.); malarabi@ksu.edu.sa (M.A.); faljamaan@ksu.edu.sa (F.A.); kalhasan@ksu.edu.sa (K.A.); falshahrani1@ksu.edu.sa (F.A.); drhbooob@gmail.com (A.A.); nsalharbi@ksu.edu.sa (N.S.A.); alrabiaah@KSU.EDU.SA (A.A.); amrjamal@ksu.edu.sa (A.J.); aleyadhy@ksu.edu.sa (A.A.-E.); fzamil@KSU.EDU.SA (F.A.); salsubaie@KSU.EDU.SA (S.A.); mbarry@ksu.edu.sa (M.B.); 2 Pediatric Department, King Saud University Medical City, King Saud University, Riyadh 11362, Saudi Arabia; rkbassrawi@ksu.edu.sa (R.B.); Dr.naifq@gmail.com (N.A.); 3 Prince Abdullah Ben Khaled Celiac Disease Research Chair, Department of Pediatrics, Faculty of Medicine, King Saud University, Riyadh 11362, Saudi Arabia; 4 Department of Psychiatry, College of Medicine, King Saud University Medical City, King Saud University, Riyadh 11362, Saudi Arabia; 5 Critical Care Department, King Saud University Medical City, King Saud University, Riyadh 11362, Saudi Arabia; 6 Department of Basic Medical Sciences, College of Medicine, Princess Nourah Bint Abdulrahman University, Riyadh 11564, Saudi Arabia; RAAssiri@pnu.edu.sa; 7 Division of Infectious Diseases, Department of Internal Medicine, King Saud University Medical City, King Saud University, Riyadh 11362, Saudi Arabia; 8 Department of Medicine, College of Medicine, Qassim University, Qassim 51452, Saudi Arabia; al_araj@hotmail.com; 9 Department of Medicine, Dr. Sulaiman Al Habib Medical Group, Riyadh 11643, Saudi Arabia; 10 Sharjah Institute of Medical Research, University of Sharjah, Sharjah 27272, United Arab Emirates; rhalwani@sharjah.ac.ae; 11 Department of Clinical Sciences, College of Medicine, University of Sharjah, Sharjah 27272, United Arab Emirates; 12 Department of Family and Community Medicine, King Saud University Medical City, Riyadh 11362, Saudi Arabia; 13 Pediatric Nephrology Department, Prince Sultan Military Medical City, Riyadh 11159, Saudi Arabia; 14 Department of Ob-Gyn, Dr. Abdul Rahman Al Mishari Hospital, Riyadh 12241, Saudi Arabia; Lalfarra19811@gmail.com; 15 Department of Ob-Gyn, Dr. Fatina Imran Medical Complex, Doha 233, Qatar; Almashdali@gmail.com; 16 Division of Infectious Diseases, Faculty of Medicine, University of Ottawa, Ottawa, ON K1H 8M5, Canada; 17 King Saud Medical City, Ministry of Health & Alfaisal University, Riyadh 11533, Saudi Arabia; zmemish@yahoo.com; 18 Hubert Department of Global Health, Emory University, Atlanta, GA 30322, USA; 19 Specialty Internal Medicine and Quality Department, Johns Hopkins Aramco Healthcare, Dhahran 34465, Saudi Arabia; jaltawfi@yahoo.com; 20 Infectious Disease Division, Department of Medicine, Indiana University School of Medicine, Indianapolis, IN 46202, USA; 21 Infectious Disease Division, Department of Medicine, Johns Hopkins University School of Medicine, Baltimore, MD 21218, USA

**Keywords:** COVID-19 uncertainties among HCWs, COVID-19, SARS-CoV-2, Omicron variant, worries, healthcare workers’ resilience, pandemic coping strategies

## Abstract

Background: As the SARS-CoV-2 Omicron variant emerged and spread globally at an alarming speed, healthcare workers’ (HCWs) uncertainties, worries, resilience, and coping strategies warranted assessment. The COVID-19 pandemic had a severe psychological impact on HCWs, including the development of Post-Traumatic Stress symptoms. Specific subgroups of HCWs, such as front-line and female workers, were more prone to poor mental health outcomes and difficulties facing stress. Methods: The responses to an online questionnaire among HCWs in the Kingdom of Saudi Arabia (KSA) were collected from 1 December 2021 to 6 December 2021, aiming to assess their uncertainties, worries, resilience, and coping strategies regarding the Omicron variant. Three validated instruments were used to achieve the study’s goals: the Brief Resilient Coping Scale (BRCS), the Standard Stress Scale (SSS), and the Intolerance of Uncertainty Scale (IUS)—Short Form. Results: The online survey was completed by 1285 HCWs. Females made up the majority of the participants (64%). A total of 1285 HCW’s completed the online survey from all regions in KSA. Resilient coping scored by the BRCS was negatively and significantly correlated with stress as scored by the SSS (r = −0.313, *p* < 0.010). Moreover, intolerance of uncertainty scored by the IUS positively and significantly correlated with stress (r = 0.326, *p* < 0.010). Increased stress levels were linked to a considerable drop in resilient coping scores. Furthermore, being a Saudi HCW or a nurse was linked to a significant reduction in resilient coping ratings. Coping by following healthcare authorities’ preventative instructions and using the WHO website as a source of information was linked to a considerable rise in resilient coping. Conclusions: The negative association between resilient coping and stress was clearly shown, as well as how underlying intolerance of uncertainty is linked to higher stress among HCWs quickly following the development of a new infectious threat. The study provides early insights into developing and promoting coping strategies for emerging SARS-CoV-2 variants.

## 1. Introduction

In November 2021, researchers in South Africa announced the emergence of a new variant of SARS-CoV-2 [1]. Later, the World Health Organization (WHO) designated this variant as a variant of concern and named it Omicron [2]. The appearance of a new infectious threat presented healthcare workers (HCWs) with a new source of stress and worry. HCWs are known to be prone to significant levels of job stress, and their stress levels have been related to burnout [3]. It has been suggested that in order to address this issue, a better understanding of the causes of work-related stress in HCWs is necessary [4].

Previous research has shown that the COVID-19 pandemic had a significant psychological toll on HCWs [5,6,7,8,9,10,11], including the development of Post-Traumatic Stress symptoms [12,13]. Specific subgroups within HCWs, such as front-line and female workers, were particularly vulnerable to worse mental health outcomes [14,15,16,17]. Furthermore, a study on HCWs following the spread of the SARS-CoV-2 Delta variant revealed high levels of worry [18].

HCWs have been struggling with uncertainty since the beginning of the COVID-19 pandemic [19]. This uncertainty was not limited to the possibility of infection but extended to the possible socio-economic impact of the pandemic [20]. Notably, difficulty in tolerating uncertainty may underlie the worry they experience in stressful situations [21]. Indeed, the construct of Intolerance of Uncertainty was developed to capture this tendency [22], which also contributes to the development of anxiety disorders [23]. In the context of the COVID-19 pandemic, higher intolerance of uncertainty has been found to correlate with stress and anxiety in various countries during the pandemic [24,25,26,27,28,29,30,31,32]. A similar correlation was also observed in HCWs, whose intolerance of uncertainty was correlated with their utilized coping strategies in the face of the pandemic [33]. Thus, it is crucial to study HCWs’ intolerance of uncertainty, and a vital construct for additional studies as the uncertainties of the pandemic persists. This is particularly important with the emergence of the Omicron variant, with its many unknowns appearing in the public health scene [1].

An essential contributor to the mental wellbeing of HCWs is their ability to cope with the continuing stress of the pandemic [34,35] and their perceived resilience [36,37]. This is most pertinent with the emergence of new variants that can threaten their health and lead to more stressful working environments. Most relevantly, HCWs with lower resilience and higher intolerance of uncertainty were at higher risk of developing burnout during the COVID-19 pandemic [38]. Since HCWs’ stress and subsequent burnout may increase with time during the pandemic [39], the appearance of the Omicron variant may further increase stress and burnout by lengthening the pandemic for months or years.

On 1 December 2021, The Saudi Ministry of Health (MoH) announced the first case of the Omicron variant in the country [40]. With that news present in the consciousness of many HCWs, we aimed to assess their awareness and sources of worry in relation to the Omicron variant and how such worries correlated with their perceived stress and ability to cope with stressful situations. Our goals also included exploring how intolerance of uncertainty relates to stress and whether higher resilient coping was correlated with decreased stress. By studying factors associated with HCWs’ stress and resilient coping during the first week of the WHO announcement, we hoped to shed light on the mental health of healthcare workers facing Omicron and possible future variants of COVID-19.

## 2. Method

This was a national, cross-sectional survey among HCWs in the Kingdom of Saudi Arabia (KSA) conducted between 1 December 2021 and 6 December 2021. At that period, several countries had reported infection with the new SARS-CoV-2 Omicron variant, and KSA reported only one case. HCWs were invited through a convenience sampling technique by several professional social media platforms, including WhatsApp groups, Twitter posts, and email lists. Participants were asked about their Omicron variant awareness, worry, and stress with the emergence of the quickly spreading Omicron variant and their resilience and coping strategies during the pandemic crisis. The survey was pilot-validated and electronically distributed through SurveyMonkey^©^. The questionnaire was adapted from our previously published studies on COVID-19 stress and coping, with modifications related to the new SARS-CoV-2 Omicron variant [18,41,42,43,44].

To survey consisted of four sections. The first was concerned with HCWs’ demographics included job category, age, sex, and work area. The second was on COVID-19 related factors including previous exposure to COVID-19 patients in the last three months, whether the HCW was previously infected with COVID-19 themselves, travel history to a country with the Omicron variant in the previous one month, and the COVID-19 vaccines they received. The third was on HCWs’ worry level regarding international travel and their sources of information about the SARS-CoV-2 variants. HCWs’ anxiety was also measured by asking them to self-rate their worry levels on a 5-point Likert scale, comparing their worry towards the original COVID-19 strain, the Alpha, the Delta, and the Omicron variants.

The last section of the survey contained three validated scales. In order to assess HCWs’ perceived resilience, we incorporated the Brief Resilient Coping Scale (BRCS), a reliable and valid tool for self-rated assessment of resilient coping [45]. The BRCS consists of four 5-point Likert scale items, with responses ranging from 1 = does not describe me at all, to 5 = describes me very well point. The scale items had good internal consistency in our sample (Cronbach’s Alpha = 0.85). In addition, in order to measure HCWs’ stress levels we utilized a self-report scale developed to assess stress in various circumstances and for a range of demographics. The Standard Stress Scale (SSS) is a 5-point Likert scale composed of 11 items that measure stress [46]. The scale has good psychometric properties, and had good internal consistency in our sample (Cronbach’s Alpha = 0.70). Finally, we incorporated the Intolerance of Uncertainty Scale (IUS) to quantify our respondents’ underlying intolerance of uncertainty and relate it to their stress levels [47]. The IUS-12 is a briefer form of the original scale composed of 12 (5-point Likert items). The scale has good psychometric properties, including in our sample of HCWs (Cronbach’s alpha = 0.89).

### 2.1. Data Collection

Participants were informed before starting the survey of the purpose of this study and that their participation in this research was completely voluntary. The Institutional Review Board at King Saud University approved the study (approval 21/01039/IRB).

### 2.2. Statistical Analysis

The mean and standard deviation were used to describe the continuous variables (such as age, worry level, familiarity with the different variants, resilient coping, stress, and intolerance of uncertainty), and the frequency and percentage to describe the categorically measured variables (such as gender, clinical role, and all the variable that have been scored by yes/no). The histogram and the K-S statistical test of Normality were used to assess the statistical Normality assumption of continuous variables, and Levene’s test was used to assess the homogeneity of variance statistical assumption. The reliability analysis of the measured psychometric scales was tested with Cronbach’s alpha test. The multiple response dichotomies analysis was used to analyze the multiple response variables. The Pearson’s correlations test (r) was used to assess the correlations between the metric variables. The Multivariable Linear Regression Analysis was applied to assess the statistical significance between predictors labelled as independent variables and the (HCW’s perceived stress and resilient coping scores) labelled as dependent variables, was expressed as unstandardized beta (β) coefficients with their 95% confidence intervals. The SPSS IBM statistical analysis program Version#21 (IBM Corp. Released 2012. IBM SPSS Statistics for Windows, Version 21.0. Armonk, NY, USA) was used for the statistical data analysis. The statistical significance level was considered with a *p*-value of ≤0.05.

## 3. Results

A total of 1285 HCW’s completed the online survey from all regions in KSA. Most of the HCW’s (57.6%) worked in Riyadh Capital City and the central region. Most of the participants (64%) were females and expatriates (62.3%). Their age distribution is shown in Table 1. Most of the HCW’s (49.8%) were nurses and (24.8%) were medical consultants. Almost half of the responders (50.2%) worked in Tertiary health centers.

Of the respondents, 38.3% worked in General Hospital Wards, while 11%, 7.1%, and 4.1% worked in intensive care units (ICU), emergency room (ER), and COVID-19 Isolation wards, respectively.

Table 2 displays the findings of the surveyed HCW’s experiences with COVID-19 disease. Of the HCWs, 29% reported recent contact with COVID-19 patients, while 22.3% were previously diagnosed as COVID-19 themselves. Over the past 2 years, the mean number of PCR testing performed per HCWs due to suspicion of SARS-CoV-2 infection was (3.43 + 3.2 times). Only 2.1% of the HCW’s had been to countries with identified Omicron variant spread in the last month.

**Figure 1:**
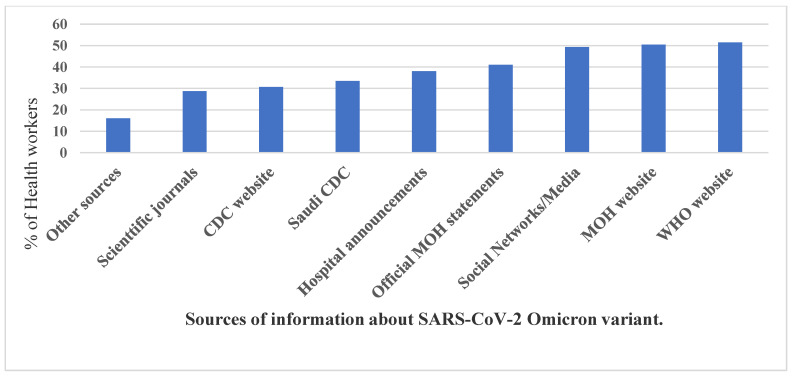
The HCWs’ used sources of information on the Omicron variant.

HCW’s were asked to self-rate their familiarity level with the Omicron and the Delta variants; the mean familiarity score was 3.24 out of 5-Likert’s points with the Omicron variant and 3.5 for the Delta variant. As shown in Figure 1, the top accessed source of information by the HCW’s was the WHO website (51.5%), followed by the Saudi MoH website (50.4%) and the Social Media channels and news (41%). In addition, 38% followed information released by formal spokespeople, and 33.5% relied on hospital announcements and the Saudi Center for Disease Control CDC website information and the US CDC website. Of the responders, 28.7% had learned about the Omicron variant from medical Journals, and 16% from other sources/channels of information. HCW’s were asked to self-rate their worry level from international travel and the various SARS-CoV-2 variants. The mean worry from travel abroad was 3.19/5 points, the worry from the original SARS-CoV-2 variant was 1.96/5 points, and from the Alpha variant was 1.67/5 points; however, the Delta variant worry level was 1.97/5 and from the Omicron variant was 2.18/5 points.

Table A1 shows the mean, standard deviation, and rank of the means for the surveyed HCW’s perceptions of their resilient coping, stress, and intolerance of uncertainty. The top perceived coping indicator according to the BRCS in our sample was the ability to grow in a positive way by handling difficulties, followed by active ways of looking to replace the losses encountered in life, then the ability to control reactions to various situations and lastly, looking for creative ways to adjust challenging situations.

**Figure 2:**
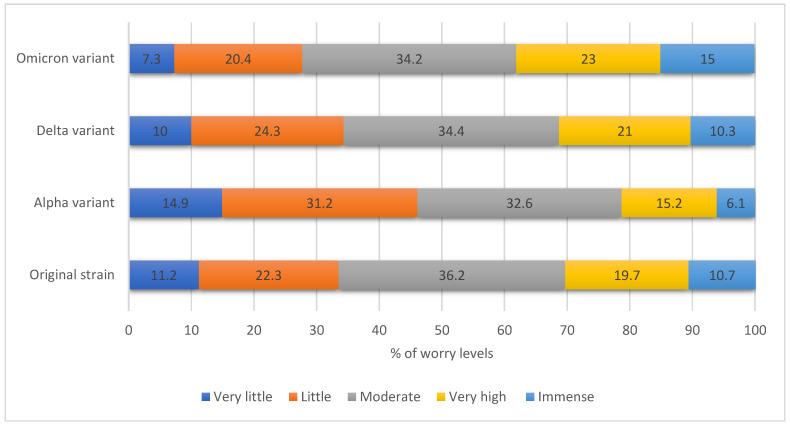
The HCWs’ perceived worry levels from various SARS-CoV-2 variants *. * Worries from original, alpha and delta strains were significantly lower than the worries from Omicron (paired samples *t*-test, *p* < 0.001 each, respectively).

The surveyed HCWs ranked their worries from original, alpha, and delta strains as significantly lower than the worries from the emerging Omicron (paired samples *t*-test, *p* < 0.001, Figure 2). Regarding the perceived stress indicators as assessed by the SSS, conducitng meaningful tasks was on the top of the list, then looking forward to the future and having people around that they could count on. Conversely, the lowest respondents’ perceived stress indicators were being afraid of how their life would look in three years, feeling exhausted after normal working days, and having a restorative sleep.

The surveyed HCW’s top perceived indicator of uncertainty, as scored by the IUS-12, was their ability to organize everything in their life ahead of time. This was followed by their agreement that one should always look ahead to avoid surprises. The third top indicator was feeling frustrated if they do not have all the information they need, and lastly, was their constant willingness to know what the future hides for them. However, the lowest-rated perceptions of uncertainty for respondents were being stopped from action by the smallest doubts then being paralyzed by uncertainty when action is needed (Table 2).

The overall mean perceived SSS score was 0.42/1 points, SD = 0.11 points, and the IUS-12 score was 31.81/60 points, SD = 8.52 points. The mean BRCS total score was 14.31/20 points, SD = 2.88 points. If cut-off scores were used for the BRCS, 39.6% of HCWs were considered to have low resilient coping, and only 18% were considered to have high resilient coping (Table 3).

For most of surveyed HCW’s, the risk of transmitting the infection to household contacts and the possibilities of new lockdown or disruption of normal daily life was the top perceived source of concern about the Omicron viral outbreak, followed by a new travel ban concern (Table 4). The higher risk of Omicron transmissibility was a concern for 57.1%. Overwhelmed healthcare services and lack of some equipment during the pandemic account for (46.3%) and (32.5%) of HCW’s sources of worry.

The respondents’ top-used coping method was following the disease transmission prevention guidelines (65.8%), followed by applying social distancing measures (56.6.8%). Family support and bonding were helpful for 56.5%, as well as having faith (54.6%) and focusing on work (41.8%). For (52.2%) of HCW’s, seeking reliable information about the disease is helpful for coping. However, few (12.5%) prefer to avoid reading about or discussing new COVID-19 strains.

Table 5 shows the bivariate Pearson’s correlation coefficients between measures of resilient coping, stress, intolerance of uncertainty of HCWs, and their self-reported levels of worry and familiarity with current and past variants of COVID-19. Resilient coping, as scored by the BRCS, was negatively and significantly correlated with stress as scored by the SSS (r = −0.313, *p* < 0.010). Moreover, Intolerance of Uncertainty, as scored by the IUS, correlated positively and significantly with stress (r = 0.326, *p* < 0.010). The analysis revealed that self-rated worry levels were mostly correlated significantly but weakly with IUS and SSS scores. However, self-reported levels of worry regarding different aspects of current or past variants of COVID-19 substantially correlated with each other (r > 0.50. *p*-value < 0.010).

Table 6 details the correlation between the resilient coping score as dependent variable and other relevant predictor variables. Saudis and nurses have shown significant negative correlation (Unstandardized Beta Coefficients −0.804, −0.742, *p*-value < 0.001), respectively. While the total number of individual COVID-19 PCR tests, mean self-rated familiarity with Omicron variant score, mean worry level from the original pandemic strain, mean perceived worry level from travel abroad due to Omicron new variant, use of WHO website as source of information and following prevention guidelines of healthcare authorities as coping strategy were significantly and positively correlated with the resilient coping score (refer to Table 6). The same was observed with the Mean Intolerance of Uncertainty Scale, which positively and significantly correlated with the resilient coping scores (0.043 *p* value < 0.001).

While the he Mean Standard Stress Scale (SSS) score was negatively and highly, significantly associated with resilient coping score (−7.975 *p*-value < 0.001), therefore we conducted another regression analysis between the (SSS) as dependent variable and other independent predictor variable relevant to the HCWs as shown in Table 7 to assess the variables contributing to their stress score and their relation to it.

**Figure 3:**
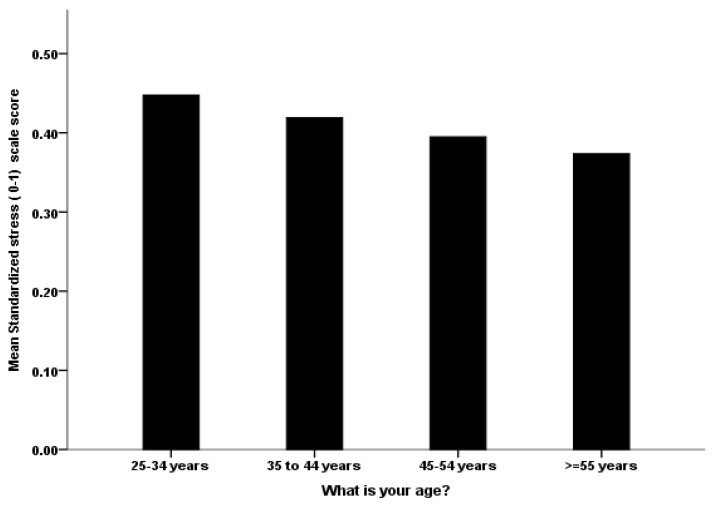
The association between the HCW’s age group with their mean perceived standardized stress score.

The HCWs’ age significantly and negatively correlated with the stress score (Unstandardized Beta Coefficients −0.017 *p* value < 0.001, Table 7 Figure 3). Other variables that also negatively and significantly correlated with HCWs’ stress score included worry level about international travel, self-rated familiarity with the delta variant, coping using sport and exercise, and utilizing hospital announcements as sources of information. Meanwhile, HCWs in the emergency room, mean worry level of the Delta variant and the original strain of SARS-CoV-2, and worry of equipment scarcity during the epidemic, all significantly and positively correlated with stress score (Table 7 for details)

## 4. Discussion

To the best of our knowledge, this is the first study to capture HCWs’ perceived stress, uncertainty, and coping in the first week of announcing the new Omicron variant. As the picture became more apparent, two weeks after the announcement of Omicron as a variant of concern by the WHO, there was a substantial public panic evident by economic volatility and major anticipation and distress amongst HCWs and healthcare policymakers around the globe [48]. Preliminary data suggested that it might be a more virulent variant, which could potentially lead to a surge in infection and multiple community and household outbreaks. This resulted in calls for declaring extreme caution as required and suggesting travel restrictions to be imposed. The situation also prompted a race to speed up booster immunization programs and revived efforts to address vaccine inequities [49].

Our sample of HCWs was large, and, similarly to other studies among HCWs, it had more female respondents [39,43]. This could reflect a higher proportion of female HCWs among the COVID-19 front lines. Women account for 75% of all HCWs worldwide, and they have been disproportionately affected by the pandemic [50]. Furthermore, half of the participants were nurses, with 62.3% being expatriates, making our sample similar to the Saudi Arabian HCW structure, which includes physicians (36.2%) and nurses (63.8%) [51]. Expatriate female nurses in KSA account for most of the nursing workforce, highlighting the need to seek their input and worries during such a crisis, especially while facing international travel restrictions [43,52].

The majority of the HCWs surveyed were frontline HCWs involved in direct patient care, hence the high rate of exposure to COVID-19 positive patients, numbers of COVID-19 PCR tests conducted, and the high rate of prior COVID-19 PCR positivity in our sample. Since KSA opened its borders for international travel in October 2021, very few HCWs had the chance to travel abroad (2% among the HCWs surveyed). Interestingly, in contrast to a previous survey conducted among HCWs in KSA about the previous Alpha variant, in which social media was the highest reported source of information about that variant, in the Omicron setting, the most common source of information about the global and national COVID-19 situation was the WHO and Saudi health authorities, represented by the Saudi CDC and MoH [43].

The top perceived source of concern for our sampled HCWs was the risk of transmitting the infection to household members (63.2%), the risk of the possible implementation of lockdown or disruption of daily life (63.1%), and possible travel ban (61.1%). In a previous study from Saudi Arabia, HCWs were also worried about travel restrictions with the emergence of the B.1.1.7 variant [43]. The current study was conducted early on after the announcement of the Omicron variant. Since then, many countries have announced a ban on travel to and from affected countries, such as South Africa [53]. Regarding lockdown, in a previous study of the B.1.1.7 variant, approximately 53% of surveyed HCWs in KSA indicated the worry about lockdown if that variant reached the country [43]. In another study, the response to the worry about lockdown was only thought of if the delta variant would cause a second wave [18]. In the United Kingdom, a third lockdown was conducted in January 2021 to prevent excess deaths from a third wave [54].

In this study, the average resilient coping score was 14.31, equivalent to 71.6% on the BRCS (as the score ranges from 4–20). Moreover, 60.4% of studied HCWs had medium or high resilient coping when categorized by cut-off scores. This indicates that most respondents had a good resilience in coping with the evolving COVID-19 pandemic. Similarly, in a previous study, the respondents had a normal range of resilient coping with a mean BRSC score of 14.9 [33]. The average IUS-12 score was 31.8, or 53.0%, if the possible score range is changed to a percentage. In a study from Italy, high levels of prospective intolerance of uncertainty as measured by the IUS-12 was a well-recognized feature of the studied Italian HCWs [33]. This is understandable since HCWs are likely to have a tendency to be able to predict the future and relay the prognosis and outcome of disease status to their patients and families. The mean SSS score in our sample of HCWs was 0.42, from a possible range of 0–1. In a previous study from Saudi Arabia, high, moderate, and low-stress levels were found among 15.8%, 77.2%, and 7% of respondents, respectively [8].

Our findings regarding the relationship between coping and stress emphasize the importance of studying these constructs in times of crisis. This is especially true for HCWs when facing stressful situations loaded with uncertainties. Our results show that HCWs who reported better resilient coping during the first week after the announcement of the Omicron variant of COVID-19 had lower stress levels on average. A similar relationship was reported in previous studies on HCWs during the COVID-19 pandemic [36,37]. To the best of our knowledge, this report is the first to demonstrate this relationship during the first days of the emergence of a new variant, highlighting the need for stress management and psychological support for HCWs as the pandemic evolves.

This work also supports the association between intolerance of uncertainty and negative mental health outcomes, including perceived stress, as shown in numerous studies studying both HCWs and the general public during the pandemic [24,25,28,30,31,32]. Intolerance of uncertainty has been mostly associated with anxiety disorders, especially Generalized Anxiety Disorder (GAD) [55,56], but is likely a trans-diagnostic construct predisposing to many psychiatric disorders [23,57,58,59]. This suggests that a higher intolerance of uncertainty may be a significant predictor of psychological distress in HCWs during stressful and uncertain events, such as new infectious threats, as was shown by the relationship between IUS scores and stress in our sample. Our correlation analysis also indicates how worries about certain aspects of the new COVID-19 variant were substantially correlated with each other. Studying the sources of anxiety for HCWs and how they relate to each other is essential not just for the sake of their well-being, but also for the wellbeing of the patients who rely on them during the hardships of the pandemic [60].

Our attempt to explain the differences between the studied HCWs in their reported resilient coping showed that at least some of the differences could be explained by the variables included in the survey, such as clinical role. For example, previous studies have reported that nurses may be more likely to report higher stress and anxiety [14,15,61,62,63] (16–19), in addition to lower coping during the COVID-19 pandemic [36]. These findings may be skewed by the fact that nurses are mostly female. Still, they could also be explained by the fact that they spend more time on inpatient wards, provide direct patient care, and are in charge of collecting sputum for virus detection, all of which increase their risk of exposure to COVID-19 patients. Furthermore, because of their intimate proximity to patients, they may be more vulnerable to moral injury in the form of suffering, death, and ethical problems (20). Similarly, our survey demonstrates how this subgroup of HCWs is more likely to report lower resilient coping when a new variant is discovered. As was reported in a previous study, the relationship between stress and resilient coping explained some of the variability in resilient coping in HCWs [64].

The stress that HCWs reported shortly after the announcement of the Omicron variant varied in our study, and some of that variation could be explained by their underlying intolerance of uncertainty. As demonstrated in other HCWs’ samples [33,38], a higher intolerance of uncertainty is associated with higher stress. This association is likely relevant when new infectious strains are discovered since such events are often shrouded with uncertainties. Our sample’s younger age groups were significantly more likely to report higher stress. Notably, it has become a consistent finding in COVID-19 research that younger age is associated with worse mental health outcomes in the general public [65]. This finding might be attributed to their function as caregivers in families (particularly females) who provide financial and emotional support to children and the elderly. Remarkably, reporting the use of sport and exercise to cope was associated with reducing stress in our sample. This finding may suggest a role for specific healthy coping strategies to help HCWs with the stress of the pandemic, as other authors have suggested [34,64]. Notwithstanding the importance of stress intervention for all HCWs during the pandemic [66,67], our results suggest certain groups are in greater need when new strains of COVID-19 emerge. For example, special attention may be needed for emergency department staff and those worried about the lack of hospital equipment.

### Study Limitations and Strengths

This research is subject to the limitations of cross-sectional studies, including convenient sampling, recall bias, and biased response rate. While our study is among the first research efforts to explore the initial worries and coping strategies of HCWs facing the novel SARS-CoV-2 Omicron variant, HCWs’ stress and perceptions are likely to change as more data about this variant emerges over time. Moreover, HCWs’ coping strategies may differ from one setting to another, so future research could explore this further in other countries.

## 5. Conclusions

This was an early investigation of the correlates of stress and resilient coping of HCWs immediately after the emergence of the Omicron variant of COVID-19 in late 2021. This report demonstrated the inverse relationship between resilient coping and stress, and how underlying intolerance of uncertainty is associated with higher stress in HCWs shortly after the emergence of a new infectious threat. These findings can inspire further research into the mental health of HCWs as the pandemic evolves. Similarly important, our results may help inform policymakers on how to better support front-line HCWs in their struggle to perform their duties in uncertain times of new variants outbreaks.

## Figures and Tables

**Table 1:** Descriptive analysis of the 1285 HCW’s sociodemographic characteristics and professional attributes.

	Frequency	Percentage
**Gender**		
Female	822	64
Male	463	36
**Age group (in years)**		
25–34	434	33.8
35–44	477	37.1
45–54	273	21.2
≥55 years	101	7.9
**Nationality**		
Saudi	484	37.7
Expatriate	801	62.3
**Clinical Role**		
Consultant	319	24.8
Assistant Consultant/Fellow	74	5.8
Resident/Registrar	203	15.8
Nurse	640	49.8
Technician	49	3.8
**Hospital type**		
Primary healthcare center	338	26.3
Secondary hospital	302	23.5
Tertiary hospital	645	50.2
**Hospital working area**		
Intensive care units (ICU)	141	11
Emergency Room (ER)	91	7.1
Operating Room (OR)	41	3.2
COVID-19 Isolation ward	53	4.1
General ward	492	38.3
Outpatient Department (OPD)	368	28.6
Non-clinical area	99	7.7
**Region**		
Central region	740	57.6
Eastern Provinces	71	5.5
Western Provinces	120	9.3
Northern Provinces	34	2.6
Southern Provinces	320	24.9

**Table 2:** Descriptive analysis of the HCW’s experiences of COVID-19 disease, screening, and immunization.

	Frequency	Percentage
**In the past three months: Have you been in contact with COVID-19 patients?**		
No	912	71
Yes	373	29
**Were you previously diagnosed with PCR-positive COVID-19 yourself**		
No	999	77.7
Yes	286	22.3
**How many COVID-19 tests have you had since the pandemic started ^$^**		
**During the last month: Did you travel to any country where the Omicron variant has been recorded?**		
No	1258	97.9
Yes	27	2.1
**How familiar are you with the new Omicron variant ^#^**		
Extremely aware	132	10.3
Very aware	329	25.6
Somewhat aware	579	45.1
Not so aware	199	15.5
Not at all aware	46	3.6
How familiar are you with the Delta variant *		
Extremely aware	204	15.9
Very aware	452	35.2
Somewhat aware	443	34.5
Not so aware	149	11.6
Not at all aware	37	2.9
**Using a Likert rating from 1–5, How worried are you from**		
International travel *		3.19 (1.12)
The original strain that started the first pandemic *		1.96 (1.14)
The Alpha variant (that was first described in the UK) *		1.67 (1.1)
The Delta variant (that was first described in India) *		1.97 (1.13)
The new Omicron variant *		2.18 (1.14)

* Mean (SD) 3.50 (0.99); # Mean (SD) 3.24 (0.95); $ Mean (SD) 3.43 (3.20).

**Table 3:** Descriptive analysis of the HCWs’ stress, resilient coping, and intolerance of uncertainty.

	Mean	SD	Possible Score Range	Equivalent Percentage for the Mean
Standard Stress Scale (SSS) score	0.42	0.11	0–1 points	42.0
Intolerance of Uncertainty (IUS-12) score	31.81	8.52	12–60 points	53.0
Brief Resilient Coping Scale (BRCS) score	14.31	2.88	4–20 points	71.6
**Resilient coping level**	**Frequency**		**Percentage**	
Low resilient coping	509		39.6	
Medium resilient coping	541		42.1	
High resilient coping	235		18.3	

**Table 4:** Descriptive analysis of the HCW’s beliefs, attitudes, and practices concerning the Omicron variant.

Variables	Frequency	Percentage
**Sources of worries and fears about SARS-CoV-2 Omicron variant**		
Risk of taking the disease to my family and household	791	63.2
Risk of lockdown or disruption to normal daily life	790	63.1
Risk of a travel ban	765	61.1
The higher risk of possible transmission among HCWs	715	57.1
Overwhelmed healthcare services	580	46.3
Lack of some equipment during pandemic (example: ventilator shortages)	407	32.5
The risk of depletion of my hospital’s PPE	387	30.9
Other worries	97	7.7
**Your best used coping strategies with Omicron as an HCW**		
Following the prevention guidelines of healthcare authorities	824	65.8
Social distancing	709	56.6
Family support	708	56.5
Having faith	684	54.6
Seeking reliable information	654	52.2
Focusing on work	523	41.8
Sport and exercise	481	38.4
Seeking support from friends and colleagues	415	33.1
Reading	409	32.7
Staying at home	400	31.9
Avoiding reading about or discussing new SARS-CoV-2 strains	157	12.5
Other coping methods	74	5.9

**Table 5:** Bivariate Correlations between the HCW’s measured perceptions.

	BRSC	SSS	IUS-12	PCR Tests	Worry from Travel	Worry from Original Pandemic	Worry from Alpha	Worry from Delta	Worry from Omicron	Familiarity Omicron
Standard Stress Scale (SSS) score	−0.313 **									
Intolerance of Uncertainty Scale (IUS-12) score	0.071 *	0.326 **								
Number of COVID19 PCR tests received since the pandemic	0.089 **	−0.002	0.004							
Mean worry about international travel	0.211 **	−0.058 *	0.205 **	−0.087 **						
Mean worry from the original strain that started the first pandemic?	0.148 **	0.076 **	0.241 **	−0.045	0.596 **					
Mean worry from The Alpha variant (that was first described in the UK)	0.086 **	0.082 **	0.197 **	−0.083 **	0.538 **	0.692 **				
Mean worry from The Delta variant (that was first described in India)	0.134 **	0.058 *	0.187 **	−0.083 **	0.579 **	0.685 **	0.772 **			
Mean worry from the new Omicron variant	0.167 **	−0.002	0.216 **	−0.040	0.683 **	0.651 **	0.610 **	0.686 **		
Self-rated familiarity with Omicron	0.133 **	−0.129 **	0.017	0.040	0.019	−0.005	0.016	0.028	0.041	
Self-rated familiarity with Delta	0.152 **	−0.165 **	−0.019	0.054	0.028	0.032	0.018	0.082 **	0.058 *	0.694 **

**. Correlation is significant at the 0.01 level (2-tailed). *. Correlation is significant at the 0.05 level (2-tailed).

**Table 6:** Multivariate Linear Regression Analysis of the HCWs’ resilient coping.

	Unstandardized Beta Coefficients	*p*-Value
Age group	0.020	0.814
Sex = Male	−0.190	0.292
Saudi	−0.804	<0.001
Nurse	−0.742	<0.001
Had been in contact with COVID-19 Patients recently	0.240	0.132
The total number of tested COVID-19 PCR tests since the start of pandemic	0.084	<0.001
Mean self-rated familiarity with Omicron variant score	0.241	0.002
Mean perceived Worry level from travel abroad due to Omicron new variant	0.310	<0.001
Mean Worry level from the original pandemic strain	0.170	0.037
Mean Standard Stress Scale (SSS) score (0–1)	−7.975	<0.001
Mean Intolerance of Uncertainty Scale (IUS-12) score	0.043	<0.001
Coping method = Focusing on work	0.305	0.052
Coping method: Following the prevention guidelines of healthcare authorities	0.426	0.009
Coping method = Sport and exercise	0.284	0.080
Coping method = Social distancing	−0.302	0.058
Source of information = MoH website	−0.388	0.011
Source of information = WHO website	0.539	<0.001

**Table 7:** Multivariate Linear Regression Analysis of the HCWs’ stress.

	Unstandardized Beta Coefficients	*p*-Value
Age group	−0.017	<0.001
Gender = Male	−0.009	0.159
Workplace = Emergency Room	0.034	0.003
Mean worry level about international travel	−0.011	0.001
Mean worry level from the original strain	0.012	0.001
Coping method = Sport and exercise	−0.023	<0.001
Travel history to country where the Omicron variant has been recorded	0.033	0.097
Mean self-rated familiarity with delta variant	−0.014	<0.001
Mean worry level from Delta variant	0.008	0.038
Source of information = Hospital announcements (e.g., roll-ups or newsletters)	−0.024	<0.001
Source of worry = lack of some equipment during pandemic (example: ventilator shortages)	0.013	0.046

## Data Availability

Data is available from the corresponding author upon reasonable request.

## Abbreviations

BRCS	Brief Resilient Coping Scale
CDC	Centers for Disease Control and Prevention
COVID-19	Coronavirus disease 2019
HCW	healthcare workers
IUS	Intolerance of Uncertainty Scale
MoH	Ministry of Health
SARS-CoV-2	Severe acute respiratory syndrome coronavirus 2
SSS	Standard Stress Scale
WHO	World Health Organization

## Appendix A

**Table A1:** Descriptive analysis of the HCWs’ perceived resilient coping, stress, and intolerance of uncertainty.

		Mean	SD	*Rank*
	***BRIEF RESILIENT COPING SCALE (BRCS) ITEMS***			
1	I look for creative ways to alter difficult situations.	3.48	0.89	*4*
2	Regardless of what happens to me, I believe I can control my reaction to it.	3.54	0.86	*3*
3	I believe I can grow in positive ways by dealing with difficult situations.	3.73	0.85	*1*
4	I actively look for ways to replace the losses I encounter in life.	3.56	0.86	*2*
	***STANDARD STRESS SCALE (SSS) ITEMS***			
*1	If I do not enjoy doing something, I usually do not have to do it.	3.03	1.08	*7*
2	If I do not take care of things by myself, nobody handles it.	3.21	1.23	*5*
*3	I am doing meaningful tasks.	3.9	1	*1*
4	I often feel lonely.	2.34	1.16	*11*
*5	My performance is appreciated adequately.	3.25	1.06	*4*
*6	There are people I can count on.	3.41	1.03	*3*
*7	Usually I have a restorative sleep.	3.01	1.03	*8*
8	I often think about problems.	3.07	1.06	*6*
9	After a normal day I am exhausted.	2.74	1.11	*9*
10	I am afraid about what my life will be like in three years.	2.57	1.26	*10*
*11	I am looking forward to my future.	3.85	1.08	*2*
	***INTOLERANCE OF UNCERTAINTY (IUS-12)***			
1	Unforeseen events upset me greatly.	2.65	1.04	*6*
2	It frustrates me not having all the information I need.	2.84	1.03	*3*
3	Uncertainty keeps me from living a full life.	2.59	1.06	*7*
4	One should always look ahead so as to avoid surprises.	3.06	1.01	*2*
5	A small unforeseen event can spoil everything, even with the best of planning.	2.55	1.06	*8*
6	When it’s time to act, uncertainty paralyses me.	2.24	1.04	*11*
7	When I am uncertain I can’t function very well.	2.45	1.06	*10*
8	I always want to know what the future has in store for me.	2.78	1.15	*4*
9	I can’t stand being taken by surprise.	2.46	1.05	*9*
10	The smallest doubt can stop me from acting.	2.23	1.09	*12*
11	I should be able to organize everything in advance.	3.2	1.06	*1*
12	I must get away from all uncertain situations.	2.77	1.1	*5*

* Starred items are positively worded items that were reverse ordered before computing the stress scale so greater score denoted higher stress aspects. In this table they were reported without reverse coding, however.
